# Naringenin targets FimZ to regulate type I fimbriae and reduce the virulence of *Salmonella*

**DOI:** 10.3389/fcimb.2025.1649866

**Published:** 2025-12-17

**Authors:** Qingqing Meng, Guizhen Wang, Jiahui Lu, Yifan Duan, Jingyao Wen, Manli Zhang, Feng Hu, Min Rao

**Affiliations:** 1 Department of Hepatology and Gastroenterology, The first Hospital of Jilin University, Changchun, Jilin, China; 2 College of Biological and Food Engineering, Jilin Engineering Normal University, Changchun, China

**Keywords:** type I fimbriae, inflammation, *Salmonella*, FimZ, NAR

## Abstract

*Salmonella* infection severely affects the healthy development of livestock and poultry, as well as food safety and public health. The critical role of type I fimbriae (TIFs) in promoting *Salmonella* pathogenicity makes them important targets for exploring inhibitors of *Salmonella* infection. In this study, we found that naringenin (Nar) inhibited the invasion of *Salmonella* into HeLa cells but did not affect bacterial motility. Nar reduced the transcription levels of the TIF structural proteins FimA and FimH and the chaperone proteins FimC and FimD, as determined via RT–qPCR. Molecular docking and surface plasmon resonance (SPR) assays confirmed that Nar was bound to FimZ, which directly regulates the expression of TIF, resulting in a reduction in TIF formation accompanied by a decrease in biofilm formation and bacterial adhesion to cells and alleviation of the inflammatory response. *In vivo*, Nar prolonged the survival of mice infected with *Salmonella*, improved the survival rate, reduced the inflammation level and bacterial load, and significantly alleviated histopathological damage. These results provide alternative strategies and promising lead compounds for controlling *Salmonella* infection.

## Introduction

*Salmonella* is not only a significant zoonotic pathogen but also a crucial foodborne pathogen (Ijaz et al., 2021; Aleksandrowicz et al., 2023). It can infect all livestock, including chickens, cattle, and pigs, leading to acute sepsis, arthritis, gastroenteritis, and other diseases and causing considerable economic losses to the livestock and poultry breeding industry (Ijaz et al., 2021; Koyun et al., 2023; Shaji et al., 2023). More than 2500 *Salmonella* serotypes have been identified, and the number of cases of infection caused by the consumption of *Salmonella*-contaminated livestock and poultry products has increased to hundreds of millions, with millions of deaths every year worldwide (Eng et al., 2015; GBD2017 Non-Typhoidal Salmonella Invasive Disease Collaborators, 2019). The development of bacterial resistance and the prohibition of the use of antibiotics make the development of novel inhibitors of *Salmonella* infection urgent (Threlfall, 2002; Aleksandrowicz et al., 2023).

*Salmonella* type I fimbriae (TIFs) are hair-like filamentous proteins on the surface of bacteria; they have a helical structure formed by several proteins through noncovalent bonding, with a diameter of 7–100 nanometers (nm) and a length of approximately 7 micrometers (μm) (Isidro-Coxca et al., 2024) (**Supplementary Figure S1**). The protein at the tip of the TIFs, FimH, can bind to mannose on mammalian cell membranes to promote the adhesion and invasion of *Salmonella* (Grzymajlo et al., 2017; Kolenda et al., 2018), which are important for successful infection. The adhesion and invasion abilities of *Salmonella* strains that express TIFs are greater than those of strains that do not express TIFs (Trautner et al., 2008; Kuźmińska-Bajor et al., 2015). In addition, TIFs are involved in the biofilm formation of *Salmonella*, although researchers have reported that a TIF-mutant *Salmonella* Typhimurium strain does not exhibit defects in the formation of cholesterol-attached biofilms (Crawford et al., 2010). Other studies have shown that the biofilm formation of *Salmonella enterica serovar* Typhimurium on Hep-2 cells and murine intestinal epithelium depends on TIFs (Boddicker et al., 2002; Ledeboer et al., 2006; Teplitski et al., 2006; Dwyer et al., 2011; Gonzalez-Escobedo and Gunn, 2013). TIFs can promote biofilm formation and help *Salmonella* Typhimurium survive in humans; the formation of biofilms can lead to persistent infection, and these persisters cannot being killed in a timely manner by antibiotics, making the treatment of *Salmonella* Typhimurium infection more difficult (Boddicker et al., 2002). These findings suggest that TIFs play important roles in the infection and pathogenicity of *Salmonella*, making them ideal targets for the development of novel inhibitors of *Salmonella* infection. Reports of the inhibition of *Salmonella* infection via TIF targeting are rare.

FimZ can regulate the expression of TIFs directly (Clegg and Hughes, 2002; Kolenda et al., 2019); however, inhibitors of infection that target FimZ to affect TIF expression and reduce *Salmonella* virulence have not been reported. Naringenin (Nar) is a foodborne compound that can be used to treat multiple diseases, such as neurodegenerative diseases, Alzheimer’s and Parkinson’s diseases, autoimmune diseases and so on (Goyal et al., 2022; Cai et al., 2023; Sahoo et al., 2024). In this study, we revealed that Nar inhibited the invasion of host cells by SL1344 but did not affect bacterial motility. Nar decreased the transcription levels of critical TIFs-related proteins and inhibited TIF formation in the *Salmonella* Typhimurium strain SL1344 through direct binding with FimZ. Consequently, the adhesion, biofilm formation and inflammation levels mediated by SL1344 decreased significantly, and Nar ultimately protected the mice from SL1344 infection.

## Materials and methods

### Reagents, bacterial strains and growth conditions

*Salmonella* Typhimurium strain SL1344 is from a laboratory strain collection. High-glucose Dulbecco’s modified Eagle’s medium (DMEM), foetal bovine serum (FBS), trypsin, and penicillin–streptomycin solution were purchased from Sangon Biotech (Shanghai) Co., Ltd. Nar was purchased from Chengdu Herbpurify Co., Ltd. Luria–Bertani (LB) medium was obtained from Beijing Solarbio Science & Technology Co., Ltd. SL1344 was cultivated in LB medium at 37 °C with shaking. The strains and plasmids used for this work are shown in **Supplementary Table S1**.

### Cell culture

HeLa human cervical cancer cells and J774A.1 mouse mononuclear macrophages were cultured in DMEM supplemented with FBS (10%), penicillin (100 U/mL) and streptomycin (0.1 mg/mL) at 37 °C with 5% carbon dioxide.

### Adhesion and invasion assays

HeLa cells were seeded into 24-well plates (1×10^5^ cells/well) and cultured overnight. SL1344 was cocultured with various concentrations of Nar (0, 16, and 32 μg/mL) for four hours, after which the bacteria were harvested and suspended in DMEM after being washed three times with sterile phosphate-buffered saline (PBS), which was used to treat the cells (multiplicity of infection, MOI = 40) for 30 minutes. Afterwards, the culture medium was discarded, and the cells were lysed with 0.2% saponin after being washed three times with sterile PBS. After dilution, the samples were plated onto LB agar medium and cultured at 37 °C overnight to count the CFUs to investigate the effect of Nar on bacterial adhesion (Shi et al., 2022a). The ratio of adhesion was calculated according to the following formula: (CFU_n1_/CFU_n0_) ×100, where CFU_n1_ and CFU_n0_ represent the bacterial numbers obtained from samples with and without Nar treatment, respectively. For the invasion assay, HeLa cells were treated with SL1344 and various concentrations of Nar (0, 16, and 32 μg/mL) for one hour, after which the medium was discarded, and gentamicin (100 μg/mL) was used to kill the extracellular bacteria. One hour later, the cells were harvested and diluted after being washed with PBS three times. Equal-volume samples were plated onto LB agar medium and cultured overnight. The colonies were counted to analyze the effect of Nar on the invasion of SL1344 (Li et al., 2024). The relative invasion was calculated according to the following formula: (CFU_s1_/CFU_s0_) ×100, where CFU_s1_ and CFU_s0_ represent the bacterial numbers obtained from samples with and without Nar treatment, respectively.

### Antibacterial assay

The minimum inhibitory concentration (MIC) of Nar against SL1344 was determined on the basis of the American Society for Clinical and Laboratory Standards (CLSI) and methods described previously with some modifications (Lambert and Pearson, 2000; Andrews, 2001). Specifically, LB media containing a series of different concentrations of Nar (0–128 μg/mL) were prepared in a 96-well plate, SL1344 was added to reach a final concentration of 5 × 10^5^ colony-forming units per milliliter (CFUs/mL), and the plates were cultured at 37 °C for 24 hours. The minimum concentration with no bacterial growth was defined as the MIC. For the growth curve assay, various concentrations (0, 32, and 64 μg/mL) of Nar were cocultured with the logarithmic growth of SL1344, the optical density at 600 nm (OD600) was detected at the specified time points, and the effect of Nar on the growth of SL1344 was analyzed via statistical analysis.

### Cytotoxicity

HeLa and J774A.1 cells were separately seeded into 96-well plates (2 × 10^4^ cells/well) and cultured overnight. Nar (0, 32, and 64 μg/mL) was cocultured with these cells for six hours, the supernatant was mixed with an equal volume of lactate dehydrogenation agent (LDH; Beyotime, Shanghai, China), and the plates were incubated in the dark for 30 minutes after centrifugation (1000 rpm, 10 minutes) (Rho et al., 2020). Afterwards, the OD490 was determined to analyze the cytotoxicity of Nar. Cells treated with 0.1% Triton X-100 or DMEM alone were used as positive (PCs) or negative (NCs) controls, respectively.

### Immunofluorescence assay

HeLa cells were seeded into 24-well cell culture plates (5 × 10^4^ cells/well). The next day, SL1344 treated with or without Nar (32 μg/mL) was used to infect the cells (MOI = 20). After one hour, the medium was removed, the cells were washed twice with sterile PBS, and gentamicin (100 μg/mL) was added to treat the cells for one hour. The cells were subsequently fixed with 4% paraformaldehyde and blocked with 4% goat serum, then *Salmonella* antibody (1:3000, Abcam) and a goat anti-rabbit secondary antibody conjugated to Alexa Fluor 488 were used to detect extracellular *Salmonella.* After this, samples were treated with 0.2% Triton X-100 and blocked with goat serum, the intracellular *Salmonella* were examined by using *Salmonella* antibody and Alexa Fluor 594 conjugated secondary antibody. The nuclei were stained with Hoechst (Beyotime) (Li et al., 2024). Images were subsequently obtained using a fluorescence microscope (Olympus, IX83) with a 60x oil immersion lens to analyze the effect of Nar on *Salmonella* invasion. The ratio of invasion was calculated according to the following formula: (Numbers_s1_/Numbers_s0_) × 100, where Numbers_s1_ and Numbers_s0_ represent the bacterial numbers obtained from the samples with and without Nar treatment, respectively.

### Motility assay

This assay was performed on the basis of a method described previously (Cheng et al., 2017). Specifically, the density of SL1344 was adjusted to 2 × 10^8^ CFUs/mL, and 5-μL samples were dropped vertically onto soft LB agar medium (0.3%) supplemented with various concentrations of Nar (0, 32, and 64 μg/mL) and incubated at 37 °C for 12 hours. The diameters of the clones were measured to evaluate the effect of Nar on the motility of the bacteria.

### Real-time reverse transcription–polymerase chain reaction

SL1344 was cultured with different concentrations of Nar (0, 16, and 32 μg/mL) for four hours, and the bacteria were harvested after centrifugation (12,000 rpm, 5 minutes). The total RNA of each sample was obtained using a total RNA extraction kit (Sangon Biotech) following the manufacturer’s instructions. RT–PCR was carried out using reverse transcription kits (Solarbio, Beijing, China) to obtain cDNA, after which the cDNA was used as the template to perform a qPCR assay by using SYBR fluorescent reagent (KTSM1401; AlpalifeBio) (Shi et al., 2022a); gyrb was used as the housekeeping gene. The data were analyzed via the ΔΔCt method. The primers used here are shown in **Supplementary Table S2**, and the RT–qPCR conditions are shown in **Supplementary Table S3**.

### Computational biology

Molecular docking and dynamics simulations were carried out according to methods reported previously (Gao et al., 2020; Yang et al., 2021; Niu et al., 2022). Briefly, the FimZ protein structure was obtained from AlphaFold (AF-P26319-F1), and the Nar structure file was obtained from PubChem. FimZ was used as the receptor, and Nar was used as the ligand. A docking box was generated after both the acceptor and the ligand were treated with AutoDock Tools, and the docking calculation was carried out with AutoDock Vina (Trott and Olson, 2010; Tang et al., 2022). Molecular dynamics simulation was performed on the basis of the conformation obtained from the docking calculation using GROMACS version 2020.6 (Van Der Spoel et al., 2005). The binding free energy was analyzed to predict the potential binding sites during the binding process.

### Electrophoretic mobility shift assays

EMSAs were performed according to a previously described method with some modifications (Hellman and Fried, 2007). The promoter region of *fimA* was amplified by using the primers shown in **Supplementary Table S4**, and the DNA of SL1344 was used as the template. Afterwards, the DNA (5 ng) was co-incubated with recombinant FimZ protein (500 ng) with or without various concentrations of Nar in specific buffer for 30 minutes at room temperature. The samples were separated on a 6% native polyacrylamide gel, and the DNA was observed with Typhoon 7000.

### Surface plasmon resonance

This assay was carried out on a Biacore X100 system (GE Healthcare), and the sensor chips used for this system were nitrilotriacetic acid (CM5). Prior to the assay, the His antibody was captured on the sensor chips. The running buffer (PBS-P) used for this assay was filtered and degassed using 0.22-micron filters (Millipore, Billerica, MA, USA). Afterwards, samples of Flag-FimZ-His or its mutants were captured using a His-antibody-coated CM5 sensor chip with a density of approximately 1000 RU. To detect binding between Nar and FimZ, running buffer (50 mM Tris, 150 mM NaCl, 10 mM MgCl_2_, 1 mM MnCl_2_, and 5% DMSO, pH 7.5) containing Nar (1 μg/mL to 65 μg/mL) was moved through the flow cell at a flow rate of 20 μL/minute (Nguyen et al., 2015; Olaru et al., 2015). The collected data were analyzed using Biacore X100 evaluation software.

### Transmission electron microscopy

TEM was carried out according to a protocol reported previously (Cheng et al., 2018). Specifically, SL1344 treated with different concentrations of Nar (0 and 32 μg/mL) was harvested and suspended in PBS, and 10-μL samples were applied to carbon-coated copper grids and incubated for 5 minutes at room temperature. Afterwards, the bacteria were treated with 0.5% phosphotungstic acid for ten seconds after the excess liquid was removed. After drying, the samples were observed under a transmission electron microscope (AX-FNSP, Nikon) (Dingle et al., 2011).

### Enzyme-linked immunosorbent assay

J774A.1 mouse macrophages were seeded into 6-well cell culture plates (1 × 10^6^ cells/well) and cultured overnight. SL1344 with or without Nar (32 μg/mL) was used to infect cells (MOI = 20) for four hours. The supernatant was collected after centrifugation (12,000 rpm, 5 minutes). The levels of tumor necrosis factor-α (TNF-α), interleukin-1β (IL-1β) and interleukin-6 (IL-6) were detected using an ELISA kit (Sangon Biotech, Shanghai, China) to measure the ability of Nar to mitigate bacteria-mediated inflammatory responses (Tabatabaei and Ahmed, 2022).

### Biofilm inhibition

SL1344 was seeded into 96-well culture plates to a final concentration of 2 × 10^7^ CFUs/mL; after different concentrations of Nar (0, 16, and 32 μg/mL) were added, the plates were incubated statically at 30 °C for 48 hours. The medium was subsequently discarded, and the samples were dried after being washed with PBS and then treated with crystal violet (0.1%, 200 μL/well) for twenty minutes. The OD570 values were measured after the samples were treated with glacial acetic acid (33%, 150 μL/well) for fifteen minutes (Hassan et al., 2011).

### Cloning, expression and purification of proteins and mutants

The proteins used here were constructed, expressed and purified via methods described previously (Soleymani et al., 2017; Aburto et al., 2019). Briefly, the *fimZ* whole fragment of SL1344 was obtained by polymerase chain reaction (PCR), after which the fragment was digested with restriction enzymes. Subsequently, the target gene DNA was inserted into pET-28a by using T4 DNA ligase (TransGen Biotech, Beijing, China) and transferred to *Escherichia coli* BL21 (DE3) strains (TransGen Biotech, Beijing, China). Protein expression was induced with 0.2 mM isopropyl-D-1-thiogalactopyranoside (Sigma–Aldrich). The purified proteins were harvested with 200 mM imidazole (Sigma–Aldrich). The FimZ mutants were obtained with the same method. The restriction enzymes used for this assay were *Bam*HI and *Sal*I. The primers used are listed in **Supplementary Table S4**.

### Animal model

All mouse infection models used in this work followed the rules of the Laboratory Animal Ethics Committee of Jilin University. This assay was performed as previously described with some modifications (Shu et al., 2021; Sheng et al., 2023). Female BALB/c mice (approximately 20 g, aged 6–8 weeks) were obtained from Liaoning Changsheng Biotechnology Co., Ltd. Before infection, the mice were treated with 5 mg/mL streptomycin in water for three days to ensure a clean intestinal environment. For the survival assay, each mouse received 100 μL of SL1344 suspension (1 × 10^8^ CFUs/mL) by gavage, and ten mice were assigned to each group. Two hours after infection, 100 mg/kg Nar was administered to each mouse via subcutaneous injection (twice per day), and the infection group or the blank control group received an equal volume of solvent or sterile PBS. The survival of the mice was observed every day to evaluate the protective effect of Nar against infection by SL1344. For other analytical indicators, each mouse was treated with 5 × 10^6^ CFUs of bacteria via the same therapeutic method. Four days later, the blood was harvested, and the mice were euthanized (cervical dislocation) after anaesthesia via pentobarbital sodium injection (30 mg/kg). Afterwards, the liver, spleen and caecum were harvested. The clones in the liver and spleen were statistically analyzed after culture on LB agar plates for 18 hours. Serum levels of cytokines were detected to evaluate the remission effect of Nar.

### Statistical analysis

The data are presented as the means with standard deviations (SDs) of three independent experiments. Statistical analysis was carried out via an unpaired *t test* in GraphPad Prism 9.5.0. Significance was defined as p ≤ 0.05.

## Results

### Nar does not affect the growth of SL1344 but inhibits its invasion of HeLa cells

**Figure 1:**
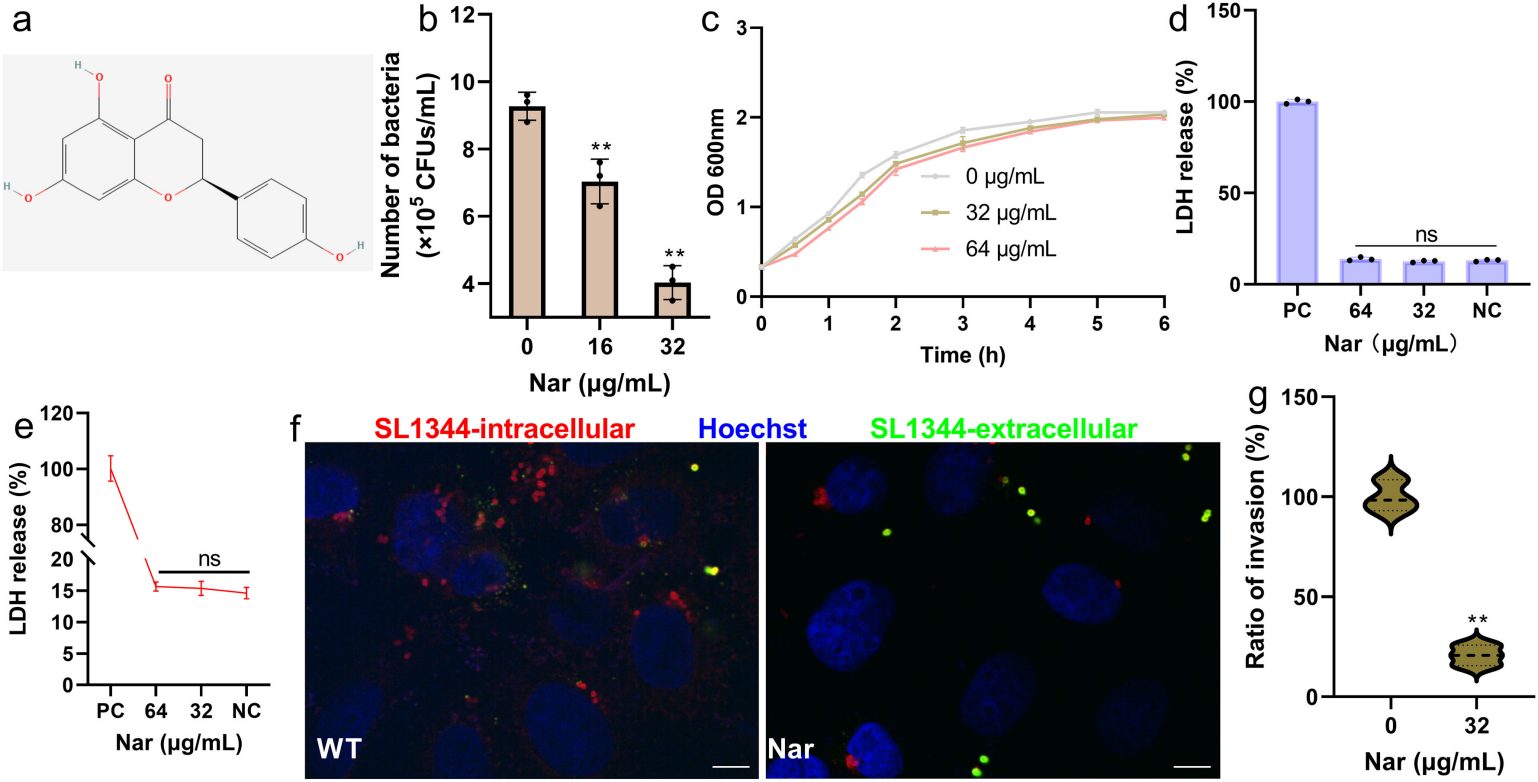
Nar inhibits the SL1344 invasion of HeLa cells. **(a)** The molecular structure of Nar. **(b)** The invasion ratio of SL1344 to HeLa cells after treatment with different concentrations of Nar. Data are shown as the means with SDs; n=3; ** p ≤ 0.01. HeLa cells were treated with SL1344 and different concentrations of Nar for one hour, the extracellular bacteria were killed with gentamicin, and the cells were lysed and coated onto LB medium to obtain colonies. **(c)** The growth trends of SL1344 under different concentrations of Nar. SL1344 was cocultured with various concentrations of Nar, and samples were obtained at the specified time points to determine their OD_600_ values. Data are shown as the means with SDs; n=3; ns represents not significant. **(d)** LDH release by HeLa or J774A.1 cells **(e)** after treatment with different concentrations of Nar. Data are shown as the means with SDs; n=3; ns represents not significant. HeLa and J774A.1 cells were treated with different concentrations of Nar for 6 hours, and the amount of LDH released into the supernatant was detected using an LDH kit. **(f)** Immunofluorescence images indicating the inhibitory effect of Nar on SL1344 invasion of HeLa cells and the quantified results **(g)**. Data are shown as the means with SDs; n=3; ** p ≤ 0.01. The scale bar represents 10 µM, and three independent assays were performed. HeLa cells were treated with SL1344 with or without Nar for one hour and then with gentamicin for one hour. Images were obtained, followed by fixation, blocking, antibody treatment and staining. The intracellular/extracellular *Salmonella* was detected by using *Salmonella* antibody and secondary antibody conjugated to Alexa Fluor 594/488.

After treatment with 16 μg/mL or 32 μg/mL Nar (**Figure 1a**), the percentage of SL1344 invading HeLa cells decreased to 75.82% and 43.41%, respectively (**Figure 1b**). The MIC of Nar against SL1344 was greater than 128 µg/mL (**Supplementary Table S5**). The growth curves revealed that Nar does not affect the growth of SL1344, as the bacteria showed similar growth when treated with or without Nar (**Figure 1c**). The amount of LDH did not differ when HeLa or J774A.1 cells received different concentrations of Nar (**Figures 1d, e**), suggesting that Nar does not have cytotoxic effects. The results of the immunofluorescence assay revealed that the number of bacteria observed in the Nar treatment groups was much lower than that in the control group (**Figures 1f, g**), further confirming the inhibitory effect of Nar against SL1344 invasion of HeLa cells. These results suggest that Nar has no antibacterial properties or cytotoxicity but significantly inhibits SL1344 invasion of HeLa cells.

### Nar reduces the transcription levels of TIF structural and chaperone proteins

**Figure 2:**
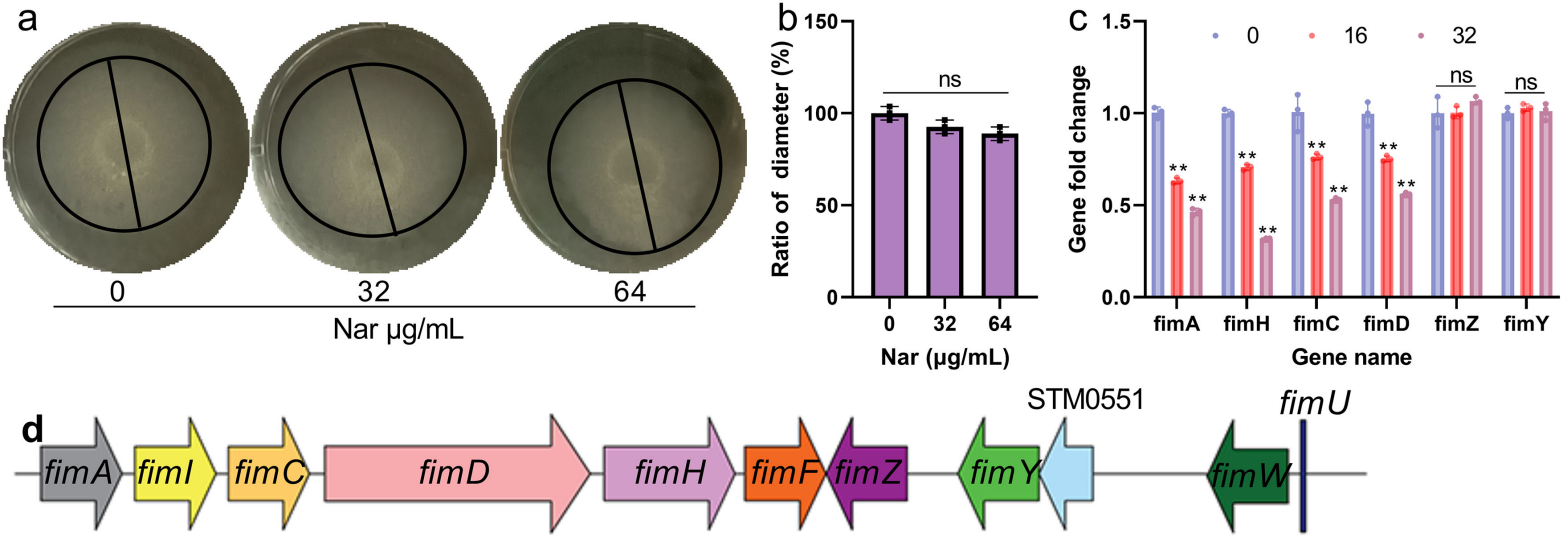
Nar does not affect the motility of SL1344 or the transportation of β-lactamase but does decrease the transcription of TIF-related proteins. **(a)** The motility of SL1344 when treated with different concentrations of Nar and the quantified results **(b)**. Data are shown as the means with SDs; n=3; ns represents not significant. SL1344 was dropped vertically onto soft LB agar medium supplemented with various concentrations of Nar and incubated at 37 °C, and the diameter of each clone was measured to evaluate the effect of Nar on the motility of the bacteria. **(c)** The transcript levels of critical TIF components and regulatory proteins under different concentrations of Nar. Data are shown as the means with SDs; n=3; ns represents not significant; ** p ≤ 0.01. SL1344 cells cultured with different concentrations of Nar were harvested and used to extract total RNA, and the expression levels of the target genes were analysed using a reverse transcription kit and SYBR fluorescent reagent. **(d)** Regulation of *Salmonella* TIF expression.

Flagella-mediated motility and TIFs affect bacterial invasion of host cells. To clarify the specific target of Nar, a motility assay was carried out, and the diameter of the samples did not significantly differ when SL1344 was treated with or without Nar (**Figures 2a, b**), indicating that invasion was not reduced by changes in the flagella. We subsequently evaluated the effect of Nar on the gene expression of TIFs by RT–qPCR, and we found that the levels of *fimA, fimH, fimC* and *fimD* decreased to 43.66%, 34.67%, 53% and 56%, respectively, when SL1344 was subjected to 32 μg/mL Nar treatment, but the levels of *fimY* and *fimZ*, which are regulatory genes, did not change (**Figures 2c, d**). These results indicate that Nar may affect TIFs to reduce the SL1344 invasion of HeLa cells.

### Nar affects TIF function by targeting FimZ

**Figure 3:**
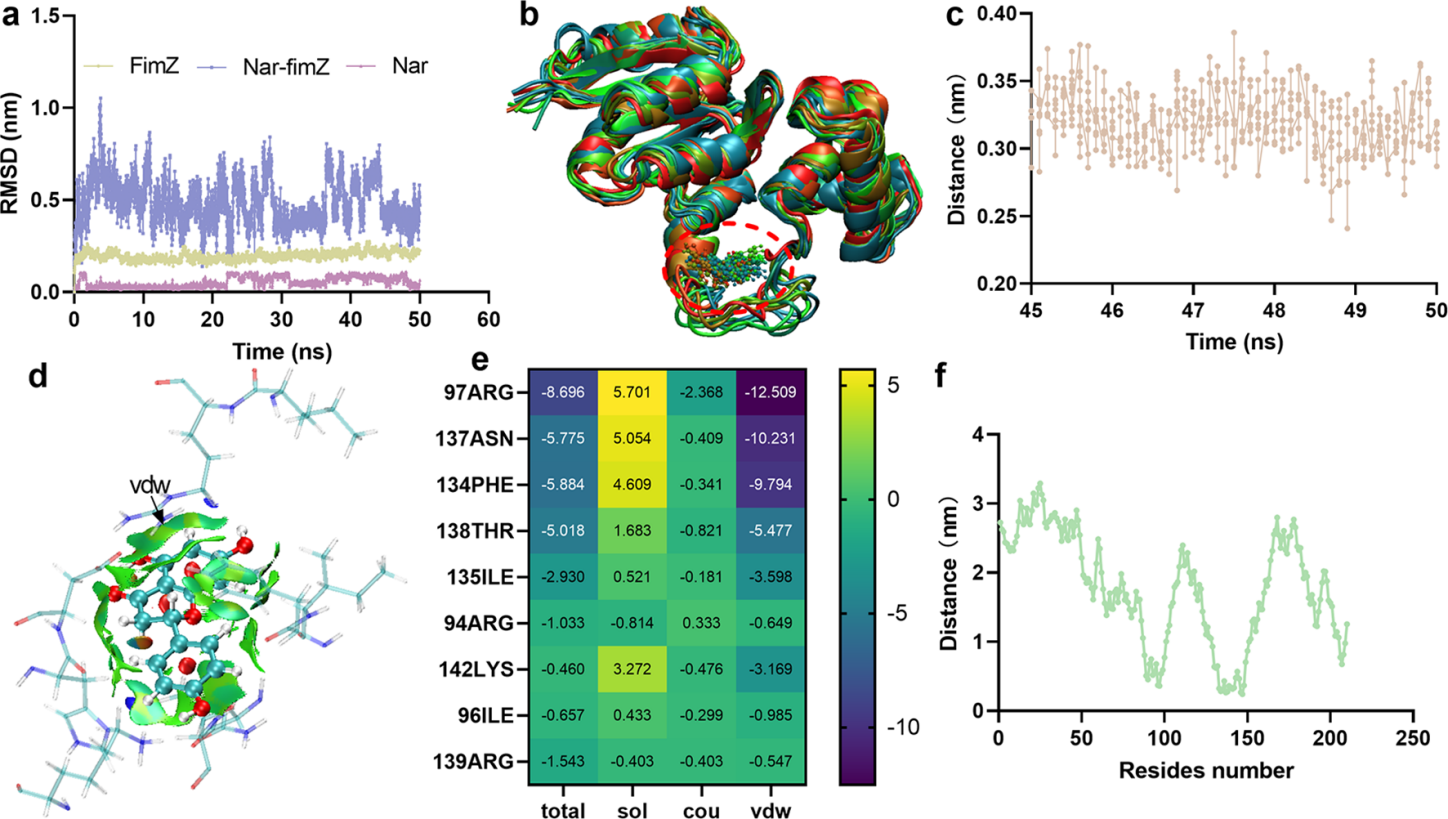
Nar binds with the critical regulatory protein FimZ. **(a)** RMSD values in the molecular simulation processes and the structural overlay **(b)**. **(c)** The distance fluctuates over the final 5 ns. **(d)** Visualization of the weak interactions between Nar and FimZ. **(e)** The energy contributions of each residue to the binding between Nar and FimZ and the distance between the residues and Nar **(f)**. FimZ was used as the receptor, Nar was used as the ligand, and AutoDock Vina was used to perform the docking calculations. GROMACS version 2020.6 was used to perform the molecular dynamics simulation assay.

The transcription levels of TIF component genes were decreased after Nar treatment, but those of regulatory genes did not change. We speculated that Nar may target regulatory proteins to affect the function of TIFs. Therefore, we performed molecular docking to explore the potential target of Nar and found that it bound to the binding pocket of FimZ with an affinity of -7.1 ± 0.39 kcal/mol. To confirm the reliability of the combination, we carried out a dynamic simulation assay and found that the RMSD values of FimZ and Nar fluctuated around 0.196 ± 0.02 nm and 0.056 ± 0.02 nm, respectively, during the simulation, which revealed that they maintained stable structures during the process (**Figure 3a**). The RMSD of Nar relative to the backbone of FimZ fluctuated around 0.485 ± 0.12 nm (**Figure 3a**), indicating that it remained in the original binding pocket, which was evidenced by the structural overlay of different frames in the trajectory (**Figure 3b**). The distance between Nar and FimZ fluctuated around 0.32 ± 0.02 nm over time (**Figure 3c**), confirming that the binding was stable. The binding free energy between Nar and FimZ was -79.08 ± 3.90 kJ/mol, which was attributed to the electrostatic force (ele; -17.74 ± 3.56 kJ/mol), van der Waals force (vdw; -133.77 ± 1.17 kJ/mol) and solvation energy (sol, 72.43 ± 2.52 kJ/mol), suggesting that vdw plays a critical role in promoting binding, which was confirmed by the results of the weak interaction analysis (**Figure 3d**). Residue energy decomposition revealed that 97ARG, 137ASN, 134PHE, 138THR, 135ILE, 94ARG, 142LYS, 96ILE, and 139ARG in FimZ contributed more energy to binding (**Figure 3e**). 97ARG, 137ASN, 134PHE and 138THR contributed more vdw and ele and presented shorter distances to Nar (**Figure 3f**). Taken together, these results indicate that vdw and ele are the main interactive forces holding Nar in the binding pocket of FimZ; this interaction originates mainly from 97ARG, 137ASN, 134PHE and 138THR.

### 97ARG, 137ASN, and 134PHE are more critical for binding

**Figure 4:**
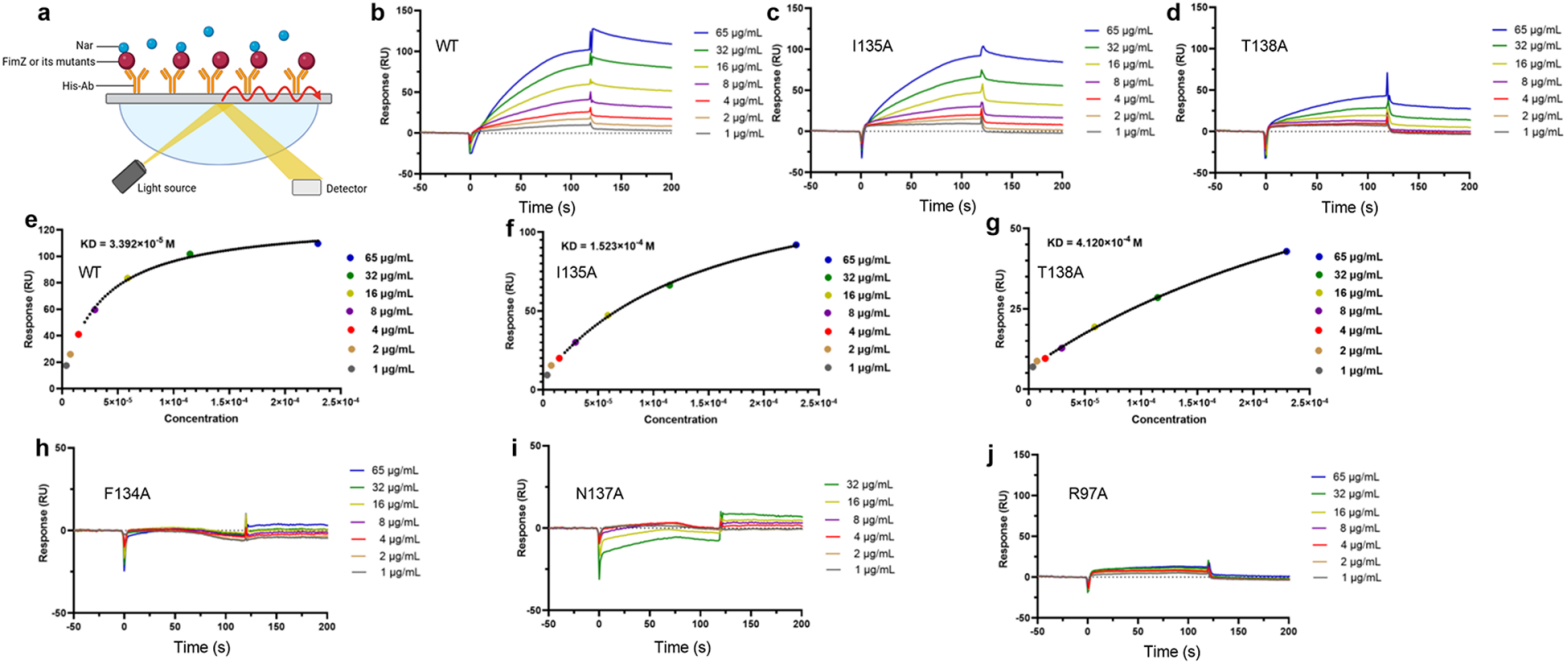
Determination of the KD between Nar and FimZ. **(a)** The theory of the SPR assay. **(b)** The response signals of WT, I135A **(c)** and T138A **(d)** when treated with various concentrations of Nar. **(e)** The fitted curves of WT, I135A **(f)** and T138A **(g)** based on their concentrations and response signals. **(h)** The response signals of F134A, N137A **(i)** and R97A **(j)**. His antibody was captured on sensor chips, and samples of Flag-FimZ-His or its mutants were captured by a His-antibody-coated CM5 sensor chip. The running buffer containing Nar (1–65 µg/mL) was moved through the flow cell to detect the binding between Nar and FimZ or its mutants, and the flow rate was 20 µL/minute.

To confirm whether the FimZ fusion protein maintained its active construct, an EMSA was carried out, and we found that the FimZ fusion protein bound to the promoter of *fimA*, but the binding was inhibited by Nar (**Supplementary Figure S2**). These results confirmed the activity of the FimZ fusion protein, and Nar inhibited its binding to the *fimA* promoter. To identify the critical binding sites, we carried out residue mutation and SPR assays (**Figure 4a**). The equilibrium dissociation constant (KD) values for the wild-type (WT), I135A and T138A strains were 3.392 × 10–^5^ mol/L, 1.523 × 10–^4^ mol/L and 4.120 × 10–^4^ mol/L, respectively (**Figures 4b–g**), suggesting that the mutation of 135ILE and 138THR affects the affinity between Nar and FimZ. However, the response signals were too weak to calculate KD values between Nar and F134A, N137A or R97A (**Figures 4h–j**). These results confirmed that ILE135, THR138, 97ARG, 137ASN and 134PHE in FimZ are important for promoting its binding to Nar, especially 97ARG, 137ASN and 134PHE.

### Nar inhibits TIF formation and reduces adhesion, biofilm formation and inflammation

**Figure 5:**
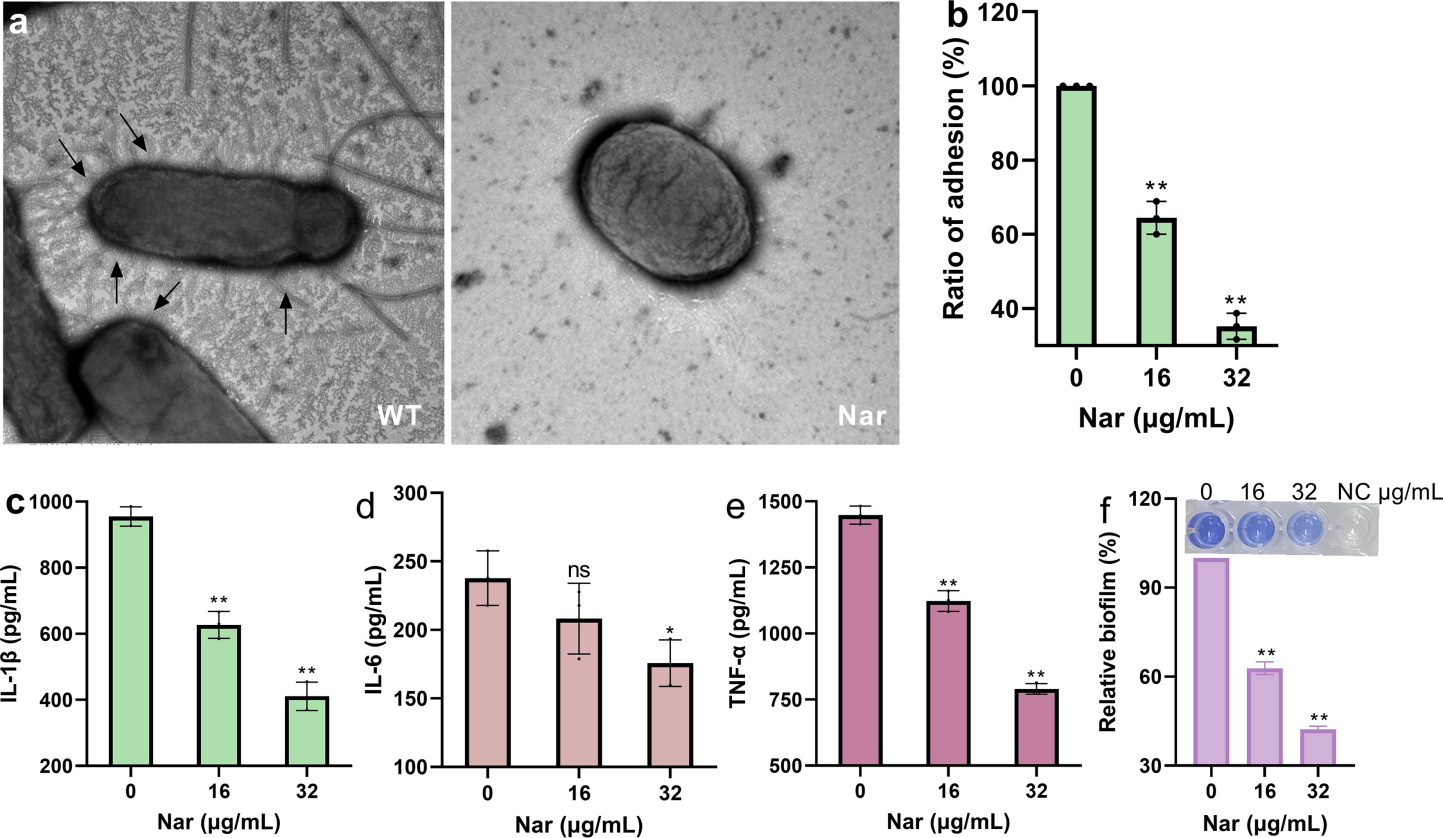
Nar reduces the adhesion effect, inflammatory response and biofilm formation by inhibiting TIF formation. **(a)** Formation of TIFs by SL1344 in the presence or absence of Nar. SL1344 cells treated with or without Nar were harvested and suspended in PBS. Afterwards, the samples were applied to carbon-coated copper grids and treated with 0.5% phosphotungstic acid, and images were obtained via transmission electron microscopy (AX-FNSP, Nikon); the acceleration voltage was 80 kV, and the scale bar was 500 nm. **(b)** The adhesion ratio of SL1344 to HeLa cells after treatment with different concentrations of Nar. Data are shown as the means with SDs; n=3; ** p ≤ 0.01. HeLa cells were treated with SL1344 and different concentrations of Nar, and the cells were lysed, coated onto LB agar medium and cultured overnight. The clones were harvested to analyse the antiadhesion effect of Nar. **(c)** The levels of IL-1β, IL-6 **(d)** and TNF-α **(e)** in J774A.1 cells treated with different concentrations of Nar. Data are shown as the means with SDs; n=3; ns represents not significant; * p ≤ 0.05; ** p ≤ 0.01. J774A.1 cells were treated with SL1344 and various concentrations of Nar for 4 hours, the supernatant was collected, and the cytokine levels were detected via ELISA. **(f)** The biofilm formation of SL1344 with different concentrations of Nar. Data are shown as the means with SDs; n=3; ** p ≤ 0.01. SL1344 was treated with different concentrations of Nar for 48 hours at 30 °C, the samples were stained with crystal violet, and the OD_570_ values were obtained after the samples were treated with glacial acetic acid.

Many TIFs formed on the SL1344 strains, while the bacteria changed to oval shapes and were sterile after receiving the 64 μg/mL Nar treatment (**Figure 5a**). The adherence of SL1344 to HeLa cells decreased to 64.43% and 35.22% when 16 μg/mL or 32 μg/mL Nar was used, respectively (**Figure 5b**). The levels of IL-1β, IL-6 and TNF-α decreased to 410.69 pg/mL, 175.70 pg/mL and 790.4 pg/mL, respectively, when each sample received 32 μg/mL Nar treatment (**Figures 5c–e**), and the formation of the biofilm was reduced to 42.24% (**Figure 5f**). These results suggest that Nar binds with FimZ to inhibit the formation of TIFs and affects the underlying phenotype.

### Nar protects mice against SL1344 infection

**Figure 6:**
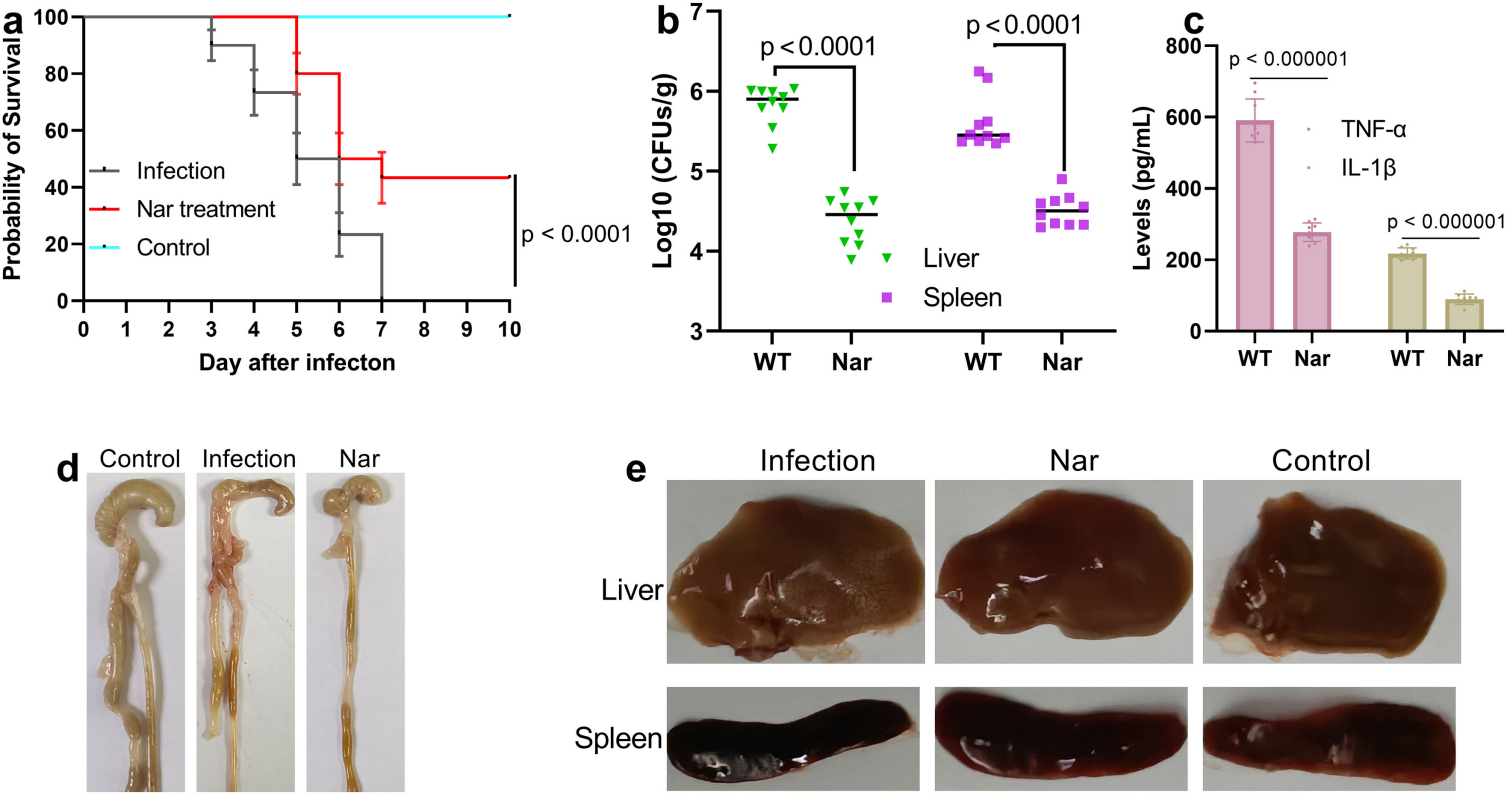
Nar protects mice against SL1344 infection. **(a)** The survival probability of mice in different groups; n=30; p<0.0001. **(b)** Logical values of the clones in the liver and spleen; n=10, p<0.0001. **(c)** Serum levels of IL-1β and TNF-α; n=9; p<0.01. **(d)** Pathologic damage to the caecum, liver and spleen **(e)** in the different groups. An SL1344 mouse infection model was constructed via gavage, and 100 mg/kg Nar was used to treat the mice after two hours of infection. The survival of the mice was observed every day to evaluate the protective effect of Nar against SL1344 infection. For other analyses, a sublethal dose of SL1344 (5 × 10^6^ CFUs/mouse) was used to construct an infection model, and the other treatments were the same as those used in the survival assay. After 48 hours of treatment, the blood was harvested, the mice were euthanized, and the liver, spleen and caecum were collected. These samples were used to analyse the bacterial burden, cytokine level and degree of tissue damage.

SL1344-infected mice that did not receive Nar treatment died three days after infection, and when the infection time increased to seven days, the survival decreased to zero. However, in the Nar treatment group, no dead mice were detected until 5 days after infection, and the final survival of this group was 43.33% (**Figure 6a**). The logarithm of the bacterial load in the liver and spleen of the mice decreased by 1.49 and 0.79, respectively, in the Nar treatment group (**Figure 6b**). The serum levels of TNF-α and IL-1β decreased by 312.95 pg/mL and 127.85 pg/mL, respectively, after treatment with Nar (**Figure 6c**). The intestinal tissues harvested from the control group showed a normal tissue status, but those from the infection group were filled with haemorrhages and were tenuous; although the intestinal tissues from the Nar treatment group did not recover to the status those of the control group, the haemorrhages disappeared, and the status was much better than that of the infection group (**Figure 6d**). In the control group, the liver and spleen samples showed normal coloration and dense tissues; samples from the infection group were swollen and less dense, and the spleen was black, whereas these symptoms were alleviated in samples from the Nar treatment group (**Figure 6e**). These results indicate that Nar can protect mice against SL1344 infection.

## Discussion

Successful *Salmonella* infection is complicated and involves motility mediated by flagella, effector protein secretion mediated by the type III secretion system, adhesion and invasion mediated by TIFs, etc (Kuźmińska-Bajor et al., 2015; Spöring et al., 2018; Westerman et al., 2021; Liu et al., 2024). As a transcriptional activator, FimZ is involved in complex gene regulatory networks; it directly regulates the gene expression of TIFs, and *fimZ* affects motility, invasion genes and biofilm formation (Baxter and Jones, 2005; Baxter and Jones, 2015). When *fimZ* was overexpressed, TIFs were formed, but the bacteria lost motility and invasion ability, suggesting that *fimZ* positively regulates TIFs (Baxter and Jones, 2005; Baxter and Jones, 2015). *Salmonella* infection can be alleviated by affecting any of these aspects. Here, we found that Nar inhibited the invasion of *Salmonella*, after which we explored the motility of the bacteria when they were exposed to Nar; however, negative feedback occurred, and we then focused our attention on TIFs.

The formation of TIFs in *Salmonella* is regulated by a strict regulatory mechanism, and three proteins (FimZ, FimY and FimW) and a tRNA encoded by *fimU* have been demonstrated to regulate the expression of TIFs. Supreet and his partner reported that FimZ and FimY can activate the promoter of *fimA* to control the expression of TIFs in an independent manner. FimW is a negative regulator of TIF formation, and its regulation is independent of FimZ, but FimY is involved in a more complicated regulatory effect by interacting with FimW on a negative feedback loop (Saini et al., 2009). In this study, when bacteria received Nar treatment, the structural genes of TIFs were downregulated, but the levels of FimZ and FimY did not significantly differ, and the formation of TIFs was inhibited. We speculate that Nar interacts with the regulator, which was confirmed by docking, molecular dynamics simulation and SPR assays. Nar bound with FimZ mainly through vdw and ele, and 97ARG, 137ASN and 134PHE in this protein were much more important for binding.

Some inhibitors of *Salmonella* have been previously reported, but many of them target the type III secretion system. Fluorothiazinon (Zigangirova et al., 2021) has been reported to be an inhibitor of the type III secretion system and to suppress *Salmonella* oral infection in mice; quercitrin (Li et al., 2023) and myricetin (Lv et al., 2021) alleviate the pathogenicity of *Salmonella enterica serovar* Typhimurium by targeting the T3SS. Fisetin (Li et al., 2024), harmine (Shi et al., 2022a), fraxetin (Shi et al., 2022b) and tannic acid (Shu et al., 2021) reduce the pathogenicity of *Salmonella* Typhimurium by affecting the function of the type III secretion system. Although the exact targets of these small molecules differ slightly, these findings provide a foundation for alternative drug development for *Salmonella* Typhimurium infection. However, inhibitors targeting TIFs to reduce *Salmonella* Typhimurium virulence are rare, especially FimZ inhibitors. Here, we confirm that Nar inhibits TIF formation by targeting the regulatory protein FimZ, thereby reducing TIF-mediated adhesion, invasion, biofilm formation, and inflammatory responses. *In vivo*, Nar has a protective effect on mice against *Salmonella* infection, but it is not potentially cytotoxic or antimicrobial, indicating its potential for combating *Salmonella* infection.

FimZ affects the expression of multiple genes, including but not limited to TIFs- and biofilm formation-related genes and *hilE* (a negative regulator gene of SPI-1). To explore other potential interaction mechanisms of Nar, we constructed a *hilE* or *fimZ* knockout strain to evaluate the effects of this phenotypic indicator. However, we encountered failure, which may have resulted in other potential mechanisms remaining undiscovered.

## Conclusion

Nar binds to the *Salmonella* TIF regulatory protein FimZ to decrease the transcription levels of structural and chaperone proteins and inhibit the formation of TIFs. Consequently, the adhesion, invasion, biofilm formation and inflammatory response mediated by TIFs were alleviated after Nar treatment. Nar has no antibacterial activity or cytotoxic effect, but it protected mice from *Salmonella* infection. These results provide new considerations and promising lead compounds for the prevention and control of *Salmonella* infection.

## Data Availability

The original contributions presented in the study are included in the article/**Supplementary Material**. Further inquiries can be directed to the corresponding author/s.
