# Supplementary material for: Naringenin targets FimZ to regulate type I fimbriae and reduce the virulence of Salmonella
Source: Front Cell Infect Microbiol. 2025 Dec 17;15:1649866. doi: 10.3389/fcimb.2025.1649866 (PMC12753884; doi:10.3389/fcimb.2025.1649866)
Supplement: Supplementary file 1 [file DataSheet1.pdf]

Supplementary files for

Naringenin targets FimZ to regulate type I fimbriae and reduce the virulence of *Salmonella*

Qingqing Meng[1#], Guizhen Wang[2], Jiahui Lu[2], Yifan Duan[2], Jingyao Wen[2], Manli Zhang[1], Feng Hu[1], Min Rao[1*]

[1]Department of Hepatology and Gastroenterology, Lequn Branch, The first Hospital of Jilin University, Changchun 130021, Jilin Province, China

[2]College of Biological and Food Engineering, Jilin Engineering Normal University, Changchun, 130052, China

***Correspondence:** Min Rao (raomin@jlu.edu.cn)

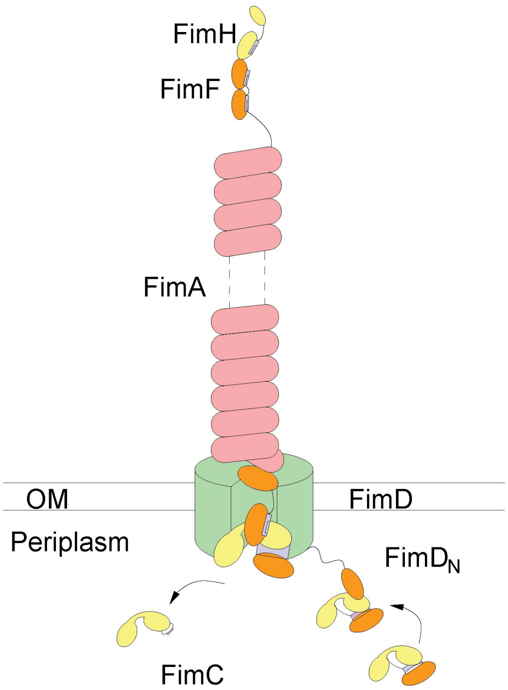

Figure S1 Components of Salmonella TIF. FimH is the protein at the tip of TIF and acts as an adhesin, binding to mannoses on the surface of mammalian cells to promote bacterial adhesion and invasion. FimA and FimF are subunit proteins; FimC is the chaperone protein of FimA, FimF, and FimH that facilitates their folding and assembly; and FimD is a transmembrane chaperone that transports proteins to the extracellular space.

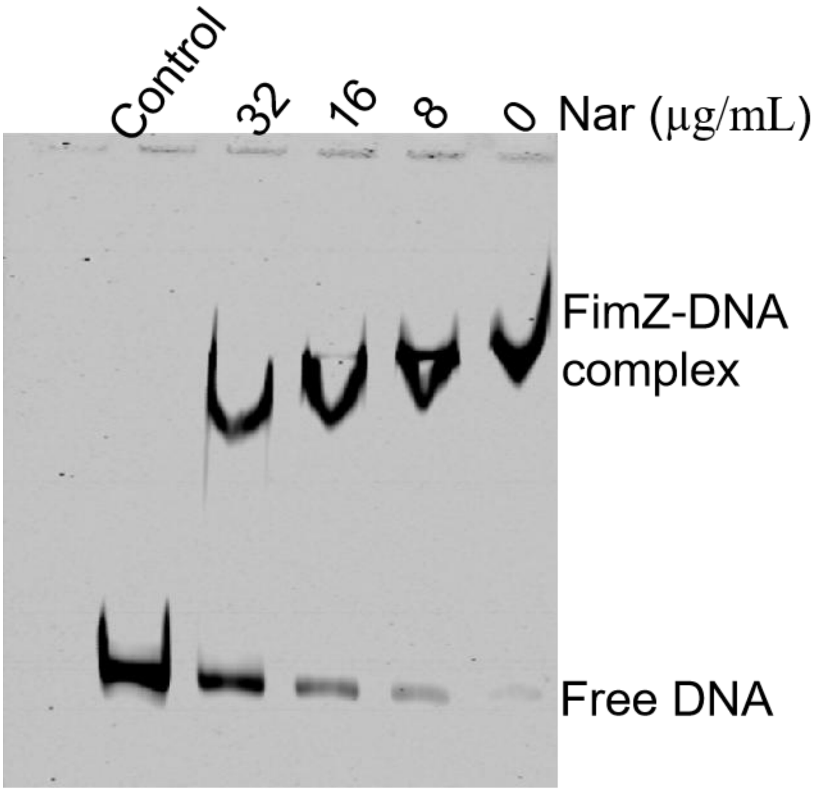

Figure S2 Nar inhibits the binding between the FimZ fusion protein and *fimA* promoter. The DNA of *fimA* promoter was co-incubated with FimZ fusion protein with or without various concentrations of Nar in the specific buffer for 30 minutes at room temperature. Then the samples were separated with 6% native polyacrylamide gel, DNA was observed with Typhoon 7000.

Table S1 The bacterial strains and plasmids used for this work

**Strains/plasmids**	**Source**
SL1344	Our laboratory strain collection
Escherichia coli DH5α	Commercial (TransGen Biotech, Beijing, China)
Escherichia coli BL21 (DE3)	Commercial (TransGen Biotech, Beijing, China)
pET-28a-fimA	Constructed in this study
pET-28a-fimH	Constructed in this study
pET-28a-SipA	Our laboratory plasmids collection
pET-28a-SipC	Our laboratory plasmids collection
SipA-TEM	Our laboratory plasmids collection

Table S2 The primers used for RT-qPCR

Primer name	Oligonucleotide (5'-3')	Length (bp)
fimA-F	ACCTCTACTATTGCGAGT	222
fimA-R	GGAGAAAGGCACCTGCGC	
fimH-F	GGCGGGGACCGCGCTCTT	214
fimH-R	TCGGTAGGTATAATTCAC	
fimC-F	GTCCGGCGAATTTGCCG	240
fimC-R	TGCGCCGTAATCATTGAC	
fimD-F	CGCGCCATCATTCCGCTA	240
fimD-R	CGCAAACGCGCCCGGCGA	
fimZ-F	GCACCGACGGCTTTACC	240
fimZ-R	GGTACGGGTATTACTGAT	
fimY-F	CGTATGGCTGGGCGTTTT	237
fimY-R	GGGAAGGTTAAGGAGGGT	
Gyrb-F	TCATTTCCACTACGAAGGCG	111
Gyrb-R	CCGAAAAAGACGGTATCGG	

Table S3 The conditions used for RT-qPCR

Category	Temperature	Time
RT-PCR	42 ℃	40 min
	85 ℃	5 min
qPCR	95 ℃	300 s
	95 ℃	20 s
	55 ℃	20 s
	72 ℃	20 s

Table S4 The primers used for cloning of proteins or EMSA assay

Primer name	Oligonucleotide (5'-3')
FimZ-F	CTGGGATCCATGAAACCTGCATCTGTTATC
FimZ-R	CTGGTCGACTTACAATAATTCGTGTGA
FimZ R97A-F	GGAAGAGCAATAGCGGCGGGCGCAAAC
FimZ R97A-R	GTTTGCGCCCGCCGCTATTGCTCTTCC
FimZ N137A-F	CTTAATTTCATCAGTGCGACCCGTACCCCCAAAG
FimZ N137A-R	CTTTGGGGGTACGGGTCGCACTGATGAAATTAAG
FimZ F134A-F	GAGACGCTTAATGCGATCAGTAATACC
FimZ F134A-R	GGTATTACTGATCGCATTAAGCGTCTC
FimZ T138A-F	CATCAGTAATGCGCGTACCCCC
FimZ T138A-R	GGGGGTACGCGCATTACTGATG
FimZ I135A-F	CGCTTAATTTCGCGAGTAATACCC
FimZ I135A-R	GGGTATTACTCGCGAAATTAAGCG
Promoter of fimA-F	ACCGCTTTACCAAAGATC
Promoter of fimA-R	CGGGTGCTTGCCTAAAGAG

The underlines represent mutation sites

Table S5 The MIC value of Nar against SL1344

**Compound**	**MIC (µg/mL)**
Nar	≥ 128
